# Supplementary material for: Topology-dependent self-structure mediation and efficient energy conversion in heat-flux-driven rotors of cholesteric droplets
Source: Nat Commun. 2018 Jan 30;9:432. doi: 10.1038/s41467-018-02910-z (PMC5789817; doi:10.1038/s41467-018-02910-z)
Supplement: Supplementary file 1 — Supplementary Information [file 41467_2018_2910_MOESM1_ESM.pdf]

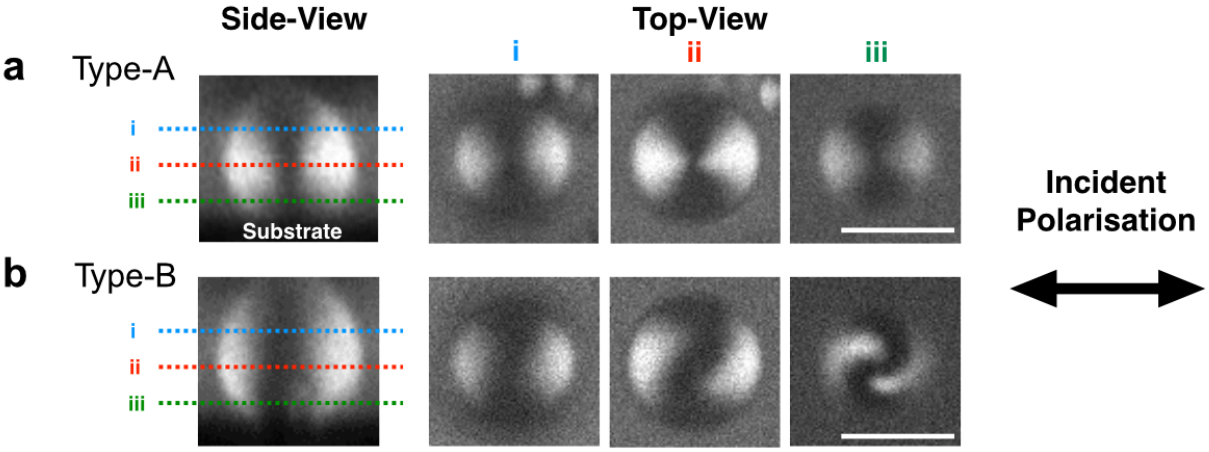

Supplementary Figure 1. **Cross-sectional confocal fluorescent micrographs taken with a linearly polarized incident light.** Top-view cross-sections for (a) Type-A and (b) Type-B taken at three different positions corresponding to i~iii in the side-view cross-sections. Type-A shows typical fluorescence microscope images for the radial nematic director field (Fig. 8a in the main text) [1]. Side-view cross-sections are taken at the vertical mid-plane, showing a spherical cap shape attached on the substrate. The light polarization is linear and parallel to the horizontal direction of the images. Scale bars, 10 μm. The concentrations of the chiral dopant are 0.2 wt% for Type-A and 0.5 wt% for Type-B. The observation is performed at RT.

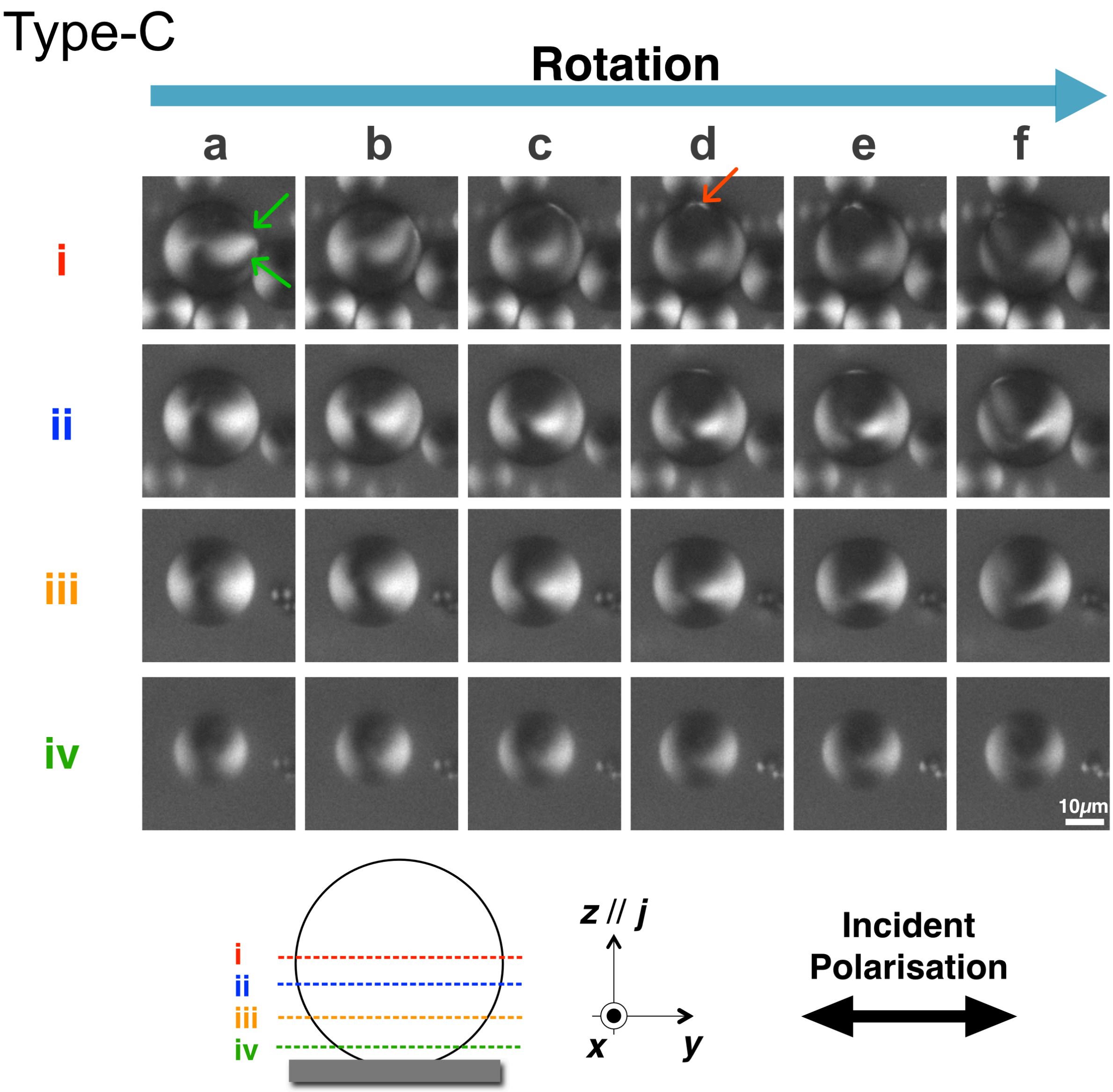

Supplementary Figure 2. **Detailed confocal fluorescence micrographs for a Type-C droplet upon the heat-flux induced rotation.** Scale bar, 10 μm. The concentration of the chiral dopant is 1.0 wt%. The stationary temperature is 32 °C. Four different cross-sectional positions (i~iv) at 3 μm intervals are chosen, corresponding to the bottom schematic. Time intervals are uncertain, because of the slow response of the confocal microscope. The green arrows in i-a indicate the dark extinction brushes, which never become bright even upon rotation. The red arrow in i-d is pointing to the strong emission from the defect at the equator.

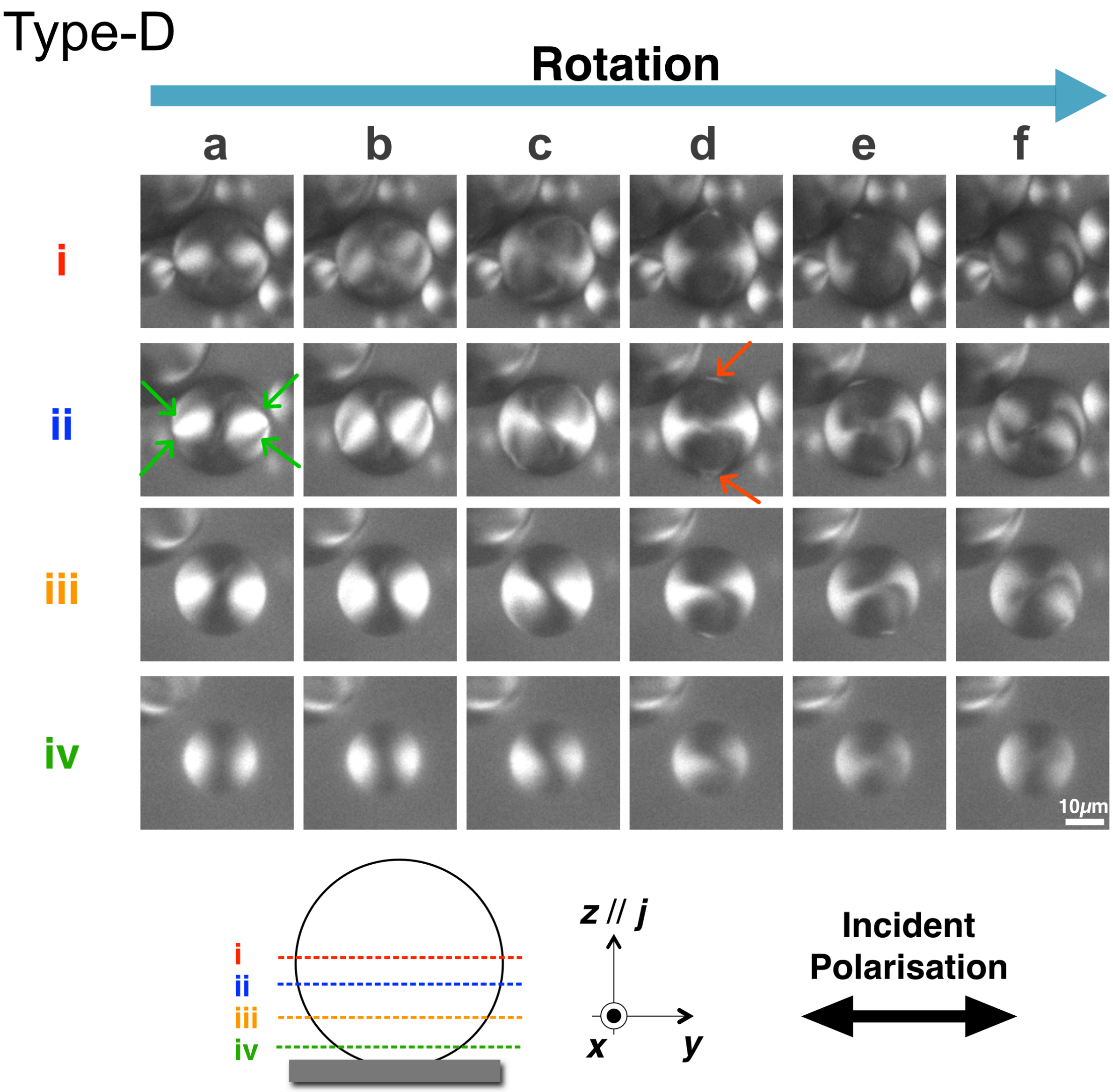

Supplementary Figure 3. Detailed confocal fluorescence micrographs for a Type-D droplet upon the heat-flux induced rotation. Scale bar, 10 μm. The concentration of the chiral dopant is 1.0 wt%. The stationary temperature is 35 °C. Four different cross-sectional positions (i~iv) at 3 μm intervals were chosen, corresponding to the bottom schematic. Time intervals were uncertain, because of the slow response of the confocal microscope. The green arrows in ii-a indicate the dark extinction brushes, which never become bright even upon rotation. The red arrows in ii-d are pointing to the strong emission from the defects at the equator.

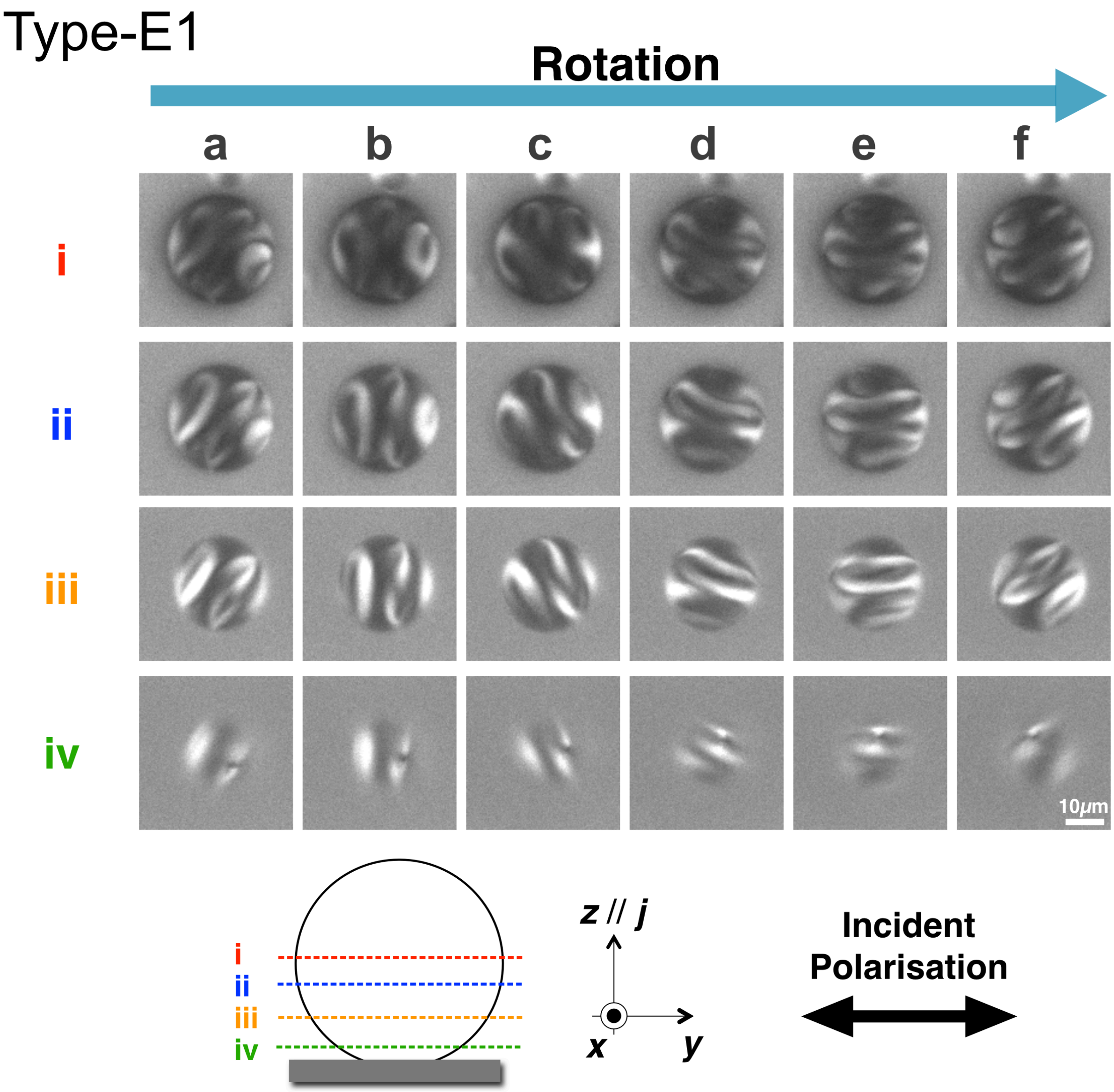

Supplementary Figure 4. Detailed confocal fluorescence micrographs for a Type-E1 droplet upon the heat-flux induced rotation. Scale bar, 10 μm. The concentration of the chiral dopant is 2.0 wt%. The stationary temperature is 33 °C. Four different cross-sectional positions (i~iv) at 3 μm intervals are chosen, corresponding to the bottom schematic. Time intervals are uncertain, because of the slow response of the confocal microscope.

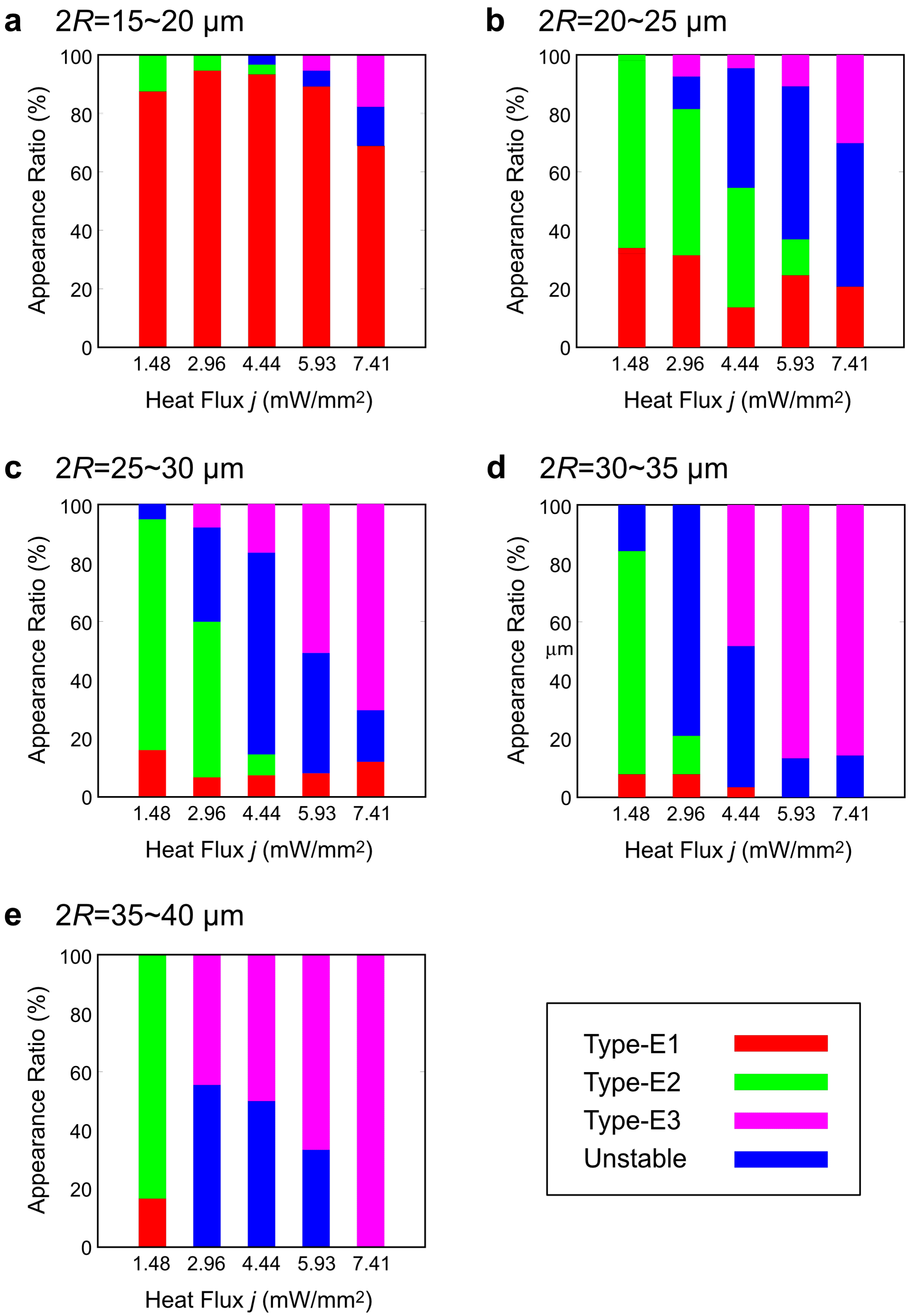

a
2R=15~20 μm
b
2R=20~25 μm
c
2R=25~30 μm
d
2R=30~35 μm
e
2R=35~40 μm
Appearance Ratio (%)
0
20
40
60
80
100
1.48
2.96
4.44
5.93
7.41
Heat Flux j (mW/mm2)
μm
Type-E1
Type-E2
Type-E3
Unstable

Supplementary Figure 5. Statistics of appearances of Type-E1, Type-E2, and Type-E3. Bar charts of appearance ratios of Type-E1, Type-E2, and Type-E3 plotted against heat-flux are sorted by droplet diameter $2R$: (a) 15–20 μm, (b) 20–25 μm, (c) 25–30 μm, (d) 30–35 μm, and (e) 35–40 μm. The concentration of the chiral dopant is 2.0 wt%, and the temperature 55°C. In smaller droplets with $2R$=15–20 μm, Type-E1 appears dominantly, almost irrespectively to the applied heal flux. In larger diameters, Type-E1 is significantly destabilized and disappears. Instead, Type-E2 appears in the lower heat flux regions. However, as the heat flux increases, Type-E2 is also destabilized but transformed into Type-E3 through Unstable (see Supplementary Movie 8). This tendency is emphasized as the droplet diameter increases.

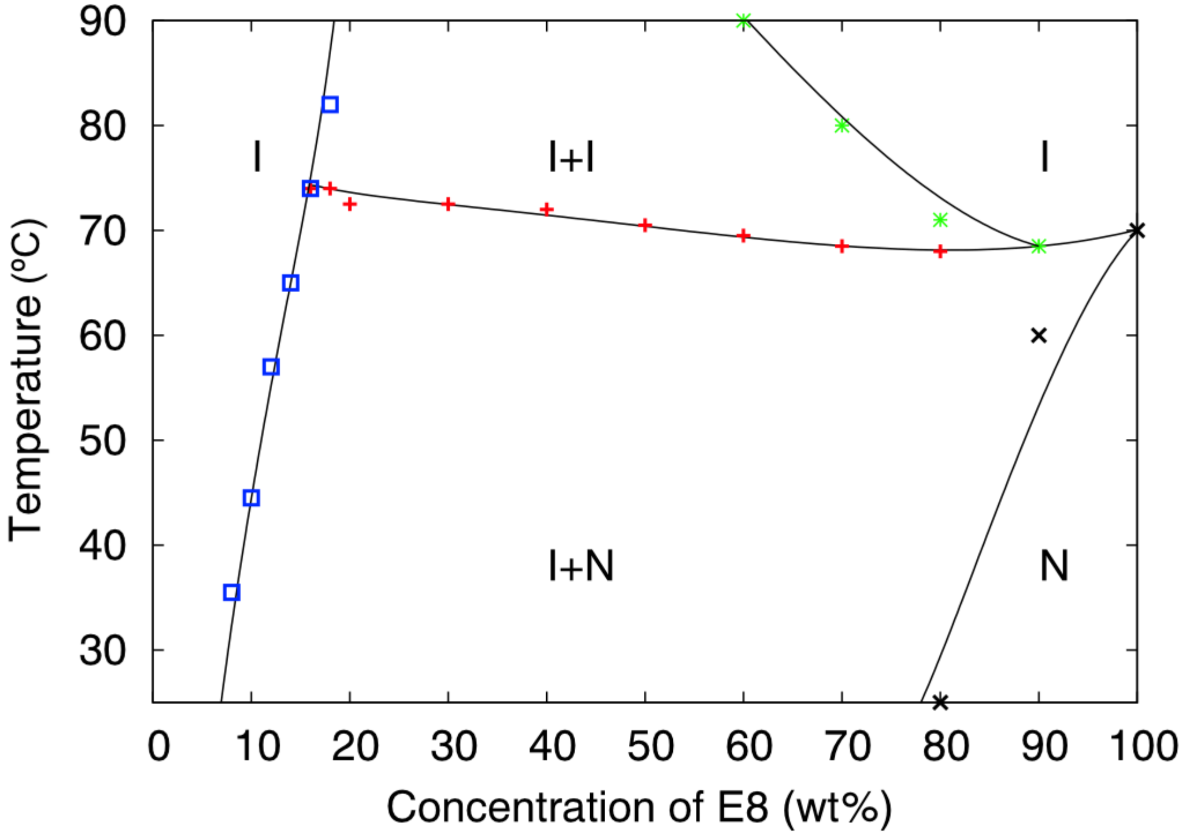

Supplementary Figure 6. Binary phase diagram of the E8-PF656 mixture system. The phase boundaries are plotted on the concentration-temperature plane. I and N represent isotropic and nematic phases, respectively. The representations of I+I and I+N are phase-segregated states of E8 in I, and PF656 in I and N, respectively. The diagram shows wide I+I and I+N regions, while they are well mixed when the concentration of E8 is smaller than ~10 wt% or larger than ~90 wt%.

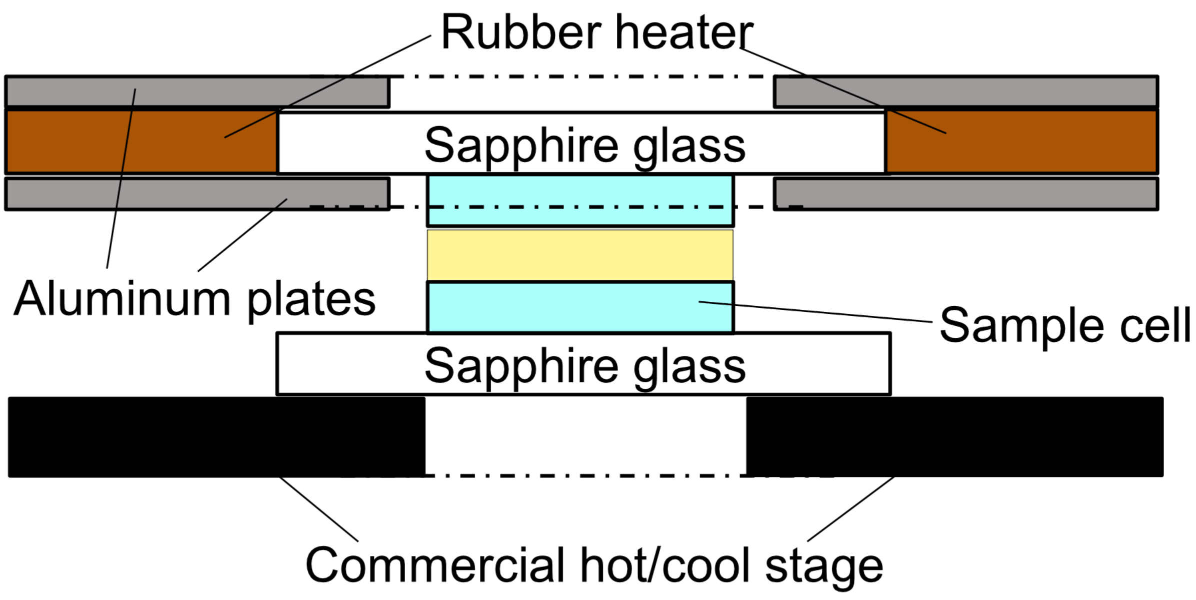

Supplementary Figure 7. Schematic of the temperature controller used in this study. The upper cell substrate is heated by a homemade hot-stage with a rubber heater, and the lower substrate is cooled by a commercial hot/cool stage (Tokai Hit). Thus, temperature gradient (hence heat flux) is introduced along the normal direction of the cell substrates. Sapphire glass plates are used as windows for optical observation, due to their excellent thermal conduction and optical transparency.

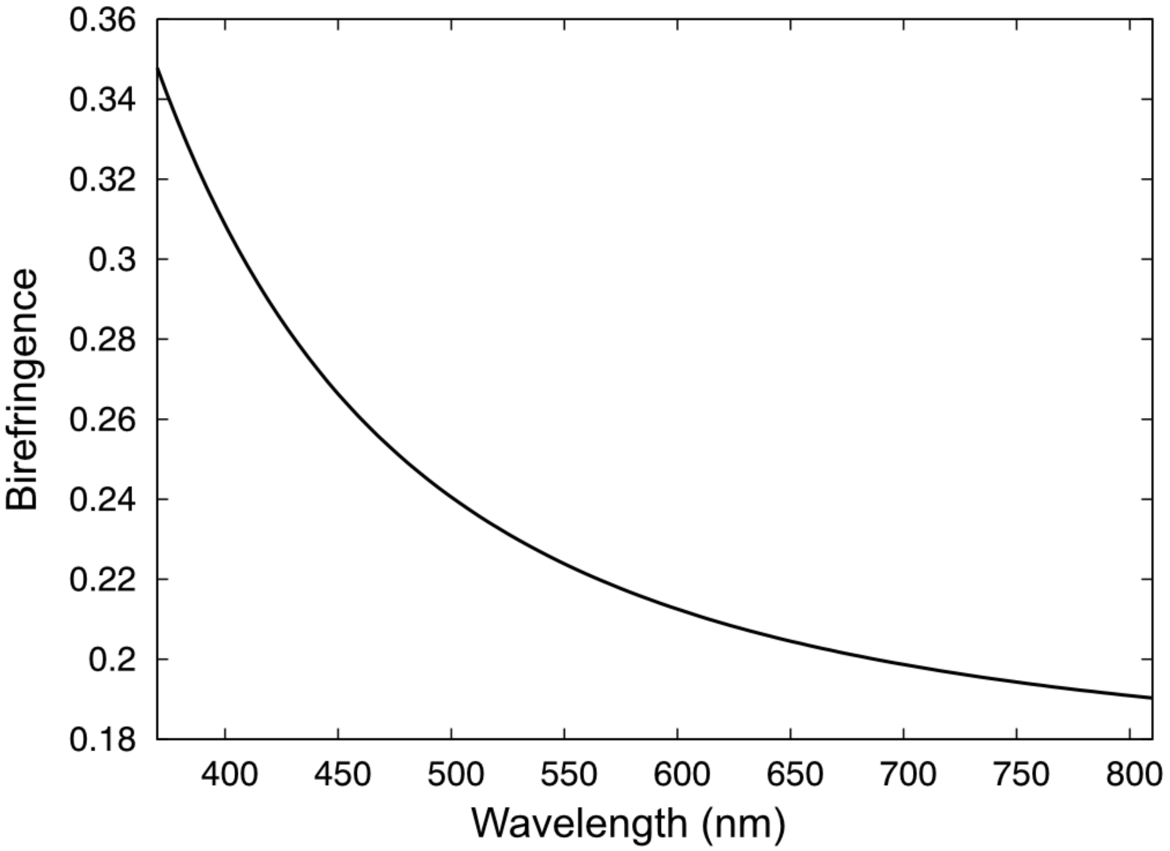

Supplementary Figure 8. Experimentally obtained dispersion curve of birefringence Δn of a mixture of E8 and PF656 in the N phase. The mixing ratio is E8:PF656 = 0.8:0.2 in weight fraction. The measured temperature is 60°C. Δn is estimated from transmission spectra obtained from a 20-μm-thick homogeneous cell using a commercial spectroscope (USB2000+VIS-NIR, Ocean Optics) [2, 3]. We use Cauchy equation $\Delta n \sim A + B/\lambda^2 + C/\lambda^4$, where λ is the optical wavelength, and A, B, and C are constants used as fitting parameters. A, B and C are determined as $A = 0.17$, $B = 8.6 \times 10^3$ nm^2, and $C = 2.1 \times 10^9$ nm^4. In the optical calculation, we use a constant refractive index of E8, n =1.53 for the ordinary light, borrowed from Supplementary Reference 4.

Supplementary Note 1. Confocal fluorescence microscopy and formulation for director fields of Type-A and Type-B: Simple radial structure and swirling structure.

As shown in Fig. 2 in the main text, Type-A emerges only for $N < \sim 2$ with a typical appearance with the extinction cross under polarization microscopy (POM), suggesting an existence of the radial symmetry around the central axis (Fig. 1a in the main text). More analytically, confocal fluorescence microscopy (CFM) was carried out using samples doped with an emissive dye (BTBP: bis-triazinyl bipyridine). Since BTBP tends to be aligned along the local liquid crystal (LC) director field, its fluorescence is pronounced in the regions where the nematic director is parallel to the polarization of the incident excitation light [5]. In addition, localisation of the dye molecules at defects works for identification of the defect positions [6, 7]. Supplementary Figure 1a is an example of the typical cross-sectional CFM images for the Type-A droplet, which clearly show a radial director distribution of Type-A (Fig. 8a in the main text). This is reasonable in the present case, because the interface between the LC and the oligomeric solvent has a homeotropic anchoring property [1]. Note that the side-view cross-sectional CFM image suggests a spherical cap shape, meaning that the droplet is slightly touching the top or bottom substrate with a finite contact angle. Actually, in the present study, this situation is common in all the five types, Type-A~E.

On the other hand, Type-B appears for $\sim 2 < N < \sim 6$. The POM image of Type-B also shows the extinction cross, but with slight swirling in a single direction, depending on the chirality (Fig. 1b in the main text). The CFM images also show deformed patterns that depend on the cross-sectional positions (Supplementary Figure 1b). These POM and CFM observations suggest that the nematic director is twisted along the viewing direction (= cell normal) and hence is not spherically but cylindrically symmetric. This reduction of symmetry is attributed to the spherical cap shape of the droplet as shown in the side-view cross-sectional CFM image. Based on these considerations, the nematic director field of Type-B can be modelled as shown in the cross-sectional schematics (Fig. 8b in the main text). We should note that both in Type-A and Type-B, owing to this structural rotational symmetry about the rotation axis, the director rotation cannot be observed by POM even if heat-flux-induced rotation (HIR) occurs.

To ensure these director models, we simulated the POM textures by conducting the Jones matrix calculation on numerically formulated structures. First, such director fields with a cylindrical symmetry can be assumed by using trial functions using cylindrical coordinates (r, ϕ, z) about

the central axis (= z-axis ∥ cell normal),

$$
\begin{aligned}
n_r &= \sin\theta\cos\left(q_c(z-R)\frac{R_\theta - r}{R_\theta}\right),\\
n_\phi &= -\sin\theta\sin\left(q_c(z-R)\frac{R_\theta - r}{R_\theta}\right),\\
n_z &= \cos\theta
\end{aligned}
\qquad (1)
$$

where q_c is taken as a constant, and

$$
\begin{aligned}
\theta &= \arctan\left(\frac{r}{z}\right),\\
R_\theta &= \sqrt{R^2 - z^2}.
\end{aligned}
\qquad (2)
$$

The factor $q_c(z-R)\frac{R_\theta - r}{R_\theta}$ represents the swirling angle in the $r\phi$ plane. The boundary condition is given by the homeotropic director orientation at the surrounding surface. When $q_c = 0$, the director field assumes a spherical radial distribution, so it is Type-A. Based on this formulation, POM images for crossed polarizers are simulated by the Jones matrix method for a uniform white light, as a mapping of optical transmission (for details, see Methods in the main text) [2, 8]. The calculated images reconstruct the POM observations well (Figs. 1h and 1i in the main text) and hence confirm the validity of our structural modelling and numerical formulation for Type-A and Type-B.

Supplementary Note 2. Confocal fluorescence microscopy and formulation for director fields of Type-C: Helical structure with a surface point defect.

Type-C is observed when $\sim 6 < N < \sim 9$, with the typical appearance of a "U-like" extinction pattern. In fact, similar droplets have also been reported in the literature [9-11]. Supplementary Figure 2 shows the CFM images of the Type-C droplet upon HIR. The cross-sectional images taken near the substrate surface (iv-a to iv-f in Supplementary Figure 2) just resemble those of Type-A, so HIR is not confirmed because of the radial symmetry. However, the images taken in the vicinity of the equatorial plane are somewhat different - a pair of dark brushes is stretched out from a certain point on the equator (indicated by green arrows in i-a in Supplementary Figure 2)

and rotates according to HIR. However, these brushes always appear to be dark irrespective of the rotation angle (i-a to ii-f in Supplementary Figure 2), suggesting that the nematic directors in these regions align in the viewing direction (= cell normal). In addition, at some angles, a bright spot appears at the connecting point of the brushes (pointed by the red arrow in i-d in Supplementary Figure 2), implying the presence of a point defect [6, 7]. The CFM images in other regions between the equatorial plane and the substrate surface show twisted dark brushes, and their connecting points are always dark and never become bright even when HIR is present. On the basis of these cross-sectional CFM images, a possible model of the director field of Type-C was proposed as shown in the schematics in Fig. 8c in the main text. Each cross-section has only one singular point away from the centre. These singular points have the vertical director orientation except at the equatorial plane at which a point defect appears on the surface (hence on the equator) (xyIII in Fig. 8c in the main text). This obviously corresponds to the bright spot in the CFM image (i-d in Supplementary Figure 2). By tracing these singular points, it can be found that they are arranged in a spiral manner, i.e., a one-dimensional helicoid around the central axis is formed in the Type-C droplet. We note again that the director at the inner surface of the droplet is fixed to the homeotropic. Thus, the director distribution of the Type-C droplet is formulated by trial functions given as Supplementary Equations (3) and (4). As is clearly seen in Fig. 1j in the main text, the simulated POM images using these trial functions reconstruct the POM image well. Of course, this simulation strongly supports the validity of our director model for Type-C.

We assumed the following trial function as the internal director field of the Type-C droplet. In the cylindrical coordinate (r, ϕ, z),

$$\begin{aligned} n_r &= \sin\theta_p \cos(\Delta\phi - \Delta\phi_s) \\ n_\phi &= \sin\theta_p \sin(\Delta\phi - \Delta\phi_s) \\ n_z &= \cos\theta_p \end{aligned} , \quad (3)$$

where θ_p, $\Delta\phi$, and ϕ_s are further expanded by the following equations.

$$
\begin{aligned}
&\theta_p = \left(1 - \exp\left(\frac{-r_1}{R_c}\right)\right)\arctan\left(\frac{\sqrt{R^2 - z^2}}{z}\right) \\
&r_1 = \sqrt{r^2 + \Delta r^2 - 2r\Delta r\cos(\phi + q_u z)} \\
&\Delta r = \begin{cases} R + z & \text{for } z < 0 \\ R - z & \text{for } z \geq 0 \end{cases} \\
&\Delta\phi = \begin{cases} \arccos\left(\dfrac{r^2 + r_1^2 - \Delta r^2}{2rr_1}\right), & \text{for } 0 < \phi + q_u z \leq \pi \\ -\arccos\left(\dfrac{r^2 + r_1^2 - \Delta r^2}{2rr_1}\right), & \text{for } -\pi < \phi + q_u z \leq 0 \end{cases} \\
&\Delta\phi_s = \begin{cases} \arccos\left(\dfrac{R_\theta^2 + R_1^2 - \Delta r^2}{2R_\theta R_1}\right), & \text{for } 0 < \phi + q_u z \leq \pi \\ -\arccos\left(\dfrac{R_\theta^2 + R_1^2 - \Delta r^2}{2R_\theta R_1}\right), & \text{for } -\pi < \phi + q_u z \leq 0 \end{cases} \\
&R_\theta = \sqrt{R^2 - z^2} \\
&R_1 = \begin{cases} -2R\cos\phi_1 & \text{for } z = 0 \\ -\Delta r\cos\phi_1 + \sqrt{R_\theta^2 - \Delta r^2 \sin^2\phi_1} & \text{for otherwise} \end{cases} \\
&\phi_1 = \begin{cases} \arccos\left(\dfrac{r^2 - r_1^2 - \Delta r^2}{2r_1\Delta r}\right), & \text{for } 0 < \phi + q_u z \leq \pi \\ -\arccos\left(\dfrac{r^2 - r_1^2 - \Delta r^2}{2r_1\Delta r}\right), & \text{for } -\pi < \phi + q_u z \leq 0 \end{cases}
\end{aligned}
\quad , \qquad (4)
$$

where R is droplet radius, q_u and R_c are constants, and $R_c << R$.

Supplementary Note 3. Confocal fluorescence microscopy and formulation for director fields of Type-D: Double-helix structure with one inner- and two surface point defects.

Type-D appears in almost the same range of N as Type-C, and a similar state has also been reported in the literature [9–11]. Supplementary Figure 3 shows the cross-sectional CFM images of the Type-D droplet upon HIR. Similarly to Type-C, the images taken in the substrate vicinity resemble that of Type-A (iv-a to iv-f in Supplementary Figure 3). On the other hand, dark figure-eight curves are observed in the regions near the equatorial plane (pointed by the green arrows in ii-a in Supplementary Figure 3), corresponding to the vertical nematic director field along the z-axis. In addition, two bright spots on the top and bottom of the "eight" are observed (pointed by red arrows in ii-d in Supplementary Figure 3), meaning that two point defects exist on the equator. Based on these observations, the internal director field of the Type-D droplet was modelled as depicted in Fig. 8d in the main text. Each cross-section has three singular points – one at the centre, and the other two arranged symmetrically on either side. By tracing these singular points, it is recognized that the outer two are arranged in a spiral manner similarly to Type-C, i.e., the double helix structure winding around the central axis in the Type-D droplet. At the equatorial plane, the outer two are exposed out to the surface and hence are located on the equator. This is consistent with the two bright spots observed in the CFM image for the equatorial plane (ii-d in Supplementary Figure 3). The formulation for Type-D is given as Supplementary Equations (5) and (6). Using optical simulations and the trial functions therein, we successfully reconstruct the POM images for Type-D (Figs. 1d and 1k in the main text).

We assumed the following trial function as the internal director field of the Type-D droplet. In the cylindrical coordinate (r, ϕ, z),

$$\begin{aligned} n_r &= \sin\theta_p \cos(\Delta\phi - \phi_e) \\ n_\phi &= \sin\theta_p \sin(\Delta\phi - \phi_e) \\ n_z &= \cos\theta_p \end{aligned} \quad , \qquad (5)$$

where θ_p, $\Delta\phi$, and ϕ_e are further expanded by the following equations.

$$\theta_p = \left(1 - \exp\left(\frac{-r}{R_c}\right)\right)\left(1 - \exp\left(\frac{-r_1}{R_c}\right)\right)\left(1 - \exp\left(\frac{-r_2}{R_c}\right)\right)\arctan\left(\frac{\sqrt{R^2 - z^2}}{z}\right)$$

$$r_i = \sqrt{r^2 + \Delta r^2 + 2r\Delta r\cos(\phi + q_e z + \pi i)} \quad (i = 1,2)$$

$$\Delta r = \begin{cases} R + z & \text{for } z < 0 \\ R - z & \text{for } z \geq 0 \end{cases}$$

$$\Delta\phi = \begin{cases} \arccos\left(\dfrac{r^2 + r_1^2 - \Delta r^2}{2rr_1}\right) & \text{for } 0 \leq |\phi + q_e z| \leq \dfrac{\pi}{2} \\ \arccos\left(\dfrac{r^2 + r_2^2 - \Delta r^2}{2rr_2}\right) & \text{for } \dfrac{\pi}{2} < |\phi + q_e z| \leq \pi \end{cases}$$

$$\phi_e = \begin{cases} \Delta\phi_{s1} + (\phi_c - \Delta\phi_{s1})\exp\left(-\dfrac{r\cos(\phi + q_e z)}{R_x}\right), & \text{for } 0 \leq |\phi + q_e z| \leq \dfrac{\pi}{2} \\ \pi - \Delta\phi_{s2} - (\phi_c - \Delta\phi_{s2})\exp\left(-\dfrac{r\cos(\phi + q_e z)}{R_x}\right), & \text{for } \dfrac{\pi}{2} < |\phi + q_e z| \leq \pi \end{cases}$$

$$\phi_c = \begin{cases} \arctan\dfrac{\Delta r}{r}, & \text{for } 0 < \phi + q_e z \leq \pi \\ -\arctan\dfrac{\Delta r}{r}, & \text{for } -\pi < \phi + q_e z \leq 0 \end{cases}$$

$$\Delta\phi_{si} = \begin{cases} \arccos\left(\dfrac{R_\theta^2 + R_i^2 - \Delta r^2}{2R_\theta R_i}\right) & \text{for } 0 < \phi + q_e z \leq \pi \\ -\arccos\left(\dfrac{R_\theta^2 + R_i^2 - \Delta r^2}{2R_\theta R_i}\right) & \text{for } -\pi < \phi + q_e z \leq 0 \end{cases}$$

$$R_\theta = \sqrt{R^2 - z^2}$$

$$R_i = \begin{cases} -2R\cos\phi_i & \text{for } z = 0 \\ -\Delta r\cos\phi_i + \sqrt{R_\theta^2 - \Delta r^2 \sin^2\phi_i} & \text{for otherwise} \end{cases}$$

$$\phi_i = \begin{cases} \arccos\left(\dfrac{r^2 - r_i^2 - \Delta r^2}{2r_i\Delta r}\right), & \text{for } 0 < \phi + q_e z \leq \pi \\ -\arccos\left(\dfrac{r^2 - r_i^2 - \Delta r^2}{2r_i\Delta r}\right), & \text{for } -\pi < \phi + q_e z \leq 0 \end{cases} \qquad (6)$$

q_e, R_c and R_x are constants, and $R_c, R_x << R$.

Supplementary Note 4. Confocal fluorescence microscopy and formulation for director fields of Type-E: Coiled defect structure.

Type-E with complex textures with streaks appears at $\sim 9 < N$. As mentioned in the previous section, this type can be further sorted into three groups, Type-E1 (Long-pitch streak pattern), Type-E2 (Short-pitch streak pattern), and Type-E3 (Concentric streak pattern). The cross-sectional CFM images of the Type-E1 droplet also show dark fringes rotating with HIR (Supplementary Figure 4). Unlike the other types, the images near the substrate also show fringes and their rotation (iv-a to iv-f in Supplementary Figure 4). This suggests that the director is periodically twisting along the direction of the fringes. Thus, it is natural to consider that a single-helix structure of the cholesteric LC is embedded and laid onto the substrate plane, where the twisting direction is defined as the 'main axis' **h**. However, still it is curious that in fact the rotating droplet never shows extinctions, i.e., the droplet shows clear fringes even at the rotation angles corresponding to the fringes being perpendicular (i-b, ii-b, iii-b, and iv-b in Supplementary Figure 4) to the incident polarisation. If the director structure is a simple helix, the fluorescence for this angle should be very weak. The existence of the second twist in the viewing direction is a plausible reason for this inconsistency. Thus we can define the 'second axis' $\mathbf{h}_s$ [12]. Actually, such a three-dimensionally twisted director configuration has already been observed in some cholesteric LC systems confined between homeotropic surfaces [13-15]. Based on these considerations, we modelled a three-dimensionally twisted structure for Type-E1 (Fig. 9a in the main text). Each cross-section only has a single singularity at the surface. Actually, this is a representation of a vortex-like topological defect winding around the droplet surface, which has already been predicted theoretically [16]. Although the presence of such a vortex is almost assured by the director distribution, the present POM and CFM images are too complicated and insufficiently clear to recognize such a defect structure.

For convenience in formulating this three-dimensional twist structure, we use the rectangular coordinates (X, Y, Z), where the Z-axis is set on the main axis **h**. Then, the director field for Type-E1 can be described by the trial functions,

$$
\begin{aligned}
n_r &= \cos\psi \cos\phi_a \\
n_\phi &= -\cos\psi \sin\phi_a , \quad (7) \\
n_z &= -\sin\psi
\end{aligned}
$$

where ψ and ϕ_a are further expanded by the following equations.

$$\psi = \begin{cases} \beta r' \sin(\phi' + qZ) + \left(\theta + \frac{\pi}{2} - \beta r' \sin(\phi' + qZ)\right)\exp\left(\frac{r' - R_\theta}{R_s}\right) & \text{for } Z < 0 \\ \beta r' \sin(\phi' + qZ) + \left(\theta - \frac{\pi}{2} - \beta r' \sin(\phi' + qZ)\right)\exp\left(\frac{r' - R_\theta}{R_s}\right) & \text{for } Z \geq 0 \end{cases}, \quad (8)$$

$$\phi_a = \phi' + qZ - (\phi' + qZ)\exp\left(\frac{r - R_\theta}{R_s}\right)\left(1 - \exp\left(\frac{-r'|\sin(\phi + qZ)|}{R_x}\right)\right)$$

where $r' = \sqrt{X^2 + Y^2}$, $\phi' = \tan^{-1}(Y/X)$, and $R_\theta' = \sqrt{R^2 - Z^2}$. q, β , R_s, and R_x are constant ($R_s, R_x \ll R$). Based on this formulation, we simulated the POM images for three different configurations of the direction of the main axis **h** with respect to the laboratory coordinates (x, y, z), i.e., $\mathbf{h} \perp \mathbf{z}$, $\mathbf{h} \parallel \mathbf{z}$, and the inclined state as schematically shown in Fig. 9c in the main text. The calculated image for $\mathbf{h} \perp \mathbf{z}$ simulates the POM of Type-E1 well (Figs. 1e and 1l in the main text), confirming the validity of the above structural model and configuration. Interestingly, the results for $\mathbf{h} \parallel \mathbf{z}$ and the inclined state (Figs. 1f and 1m in the main text) show close resemblance to the POMs of Type-E3 and Type-E2 (Figs. 1g and 1n in the main text), respectively. Thus, we conclude that the assumed director field for Type-E is basically reliable, and the textual difference among the three types of Type-E is due to the difference in the direction of the main axis **h** with respect to the z-axis.

Supplementary References

[1] OndrisCrawford, R., Boyko, E. P., Wagner, B. G., Erdmann, J. H., Žumer, S. & Doane, J. W. Microscope textures of nematic droplets in polymer dispersed liquid-crystals. *J. Appl. Phys.* **69**, 6380–6386 (1991).

[2] Kleman, M. & Lavrentovich, O. D. *Soft Matter Physics An Introduction*, (Springer, 2003).

[3] Li, J. & Wu, S. -T. Extended Cauchy equations for the refractive indices of liquid crystals. *J. Appl. Phys.* **95**, 896–901 (2004).

[4] Vaz, N. A. & Montgomery, G. P. Refractive-indexes of polymer-dispersed liquid-crystal film materials - epoxy based systems. *J. Appl. Phys.* **62**, 3161–3172 (1987).

[5] Smalyukh, I. I., Shiyanovskii, S. V. & Lavrentovich, O. D. Three-dimensional imaging of orientational order by fluorescence confocal polarizing microscopy. *Chem. Phys. Lett.* **366**, 88–96 (2001)..

[6] Ohzono, T., Katoh, K. & Fukuda, J. Fluorescence microscopy reveals molecular localisation at line defects in nematic liquid crystals. *Sci Rep.* **6**, 36477 (2016).

[7] Wang, X., Miller, D. S., Bukusoglu, E., de Pablo, J. J. & Abbott, N. L. Topological defects in liquid crystals as templates for molecular self-assembly. *Nat. Mater.* **15**, 106 (2016).

[8] Jones, R. C. A new calculus for the treatment of optical systems I. description and discussion of the calculus. *J. Opt. Soc. Am.* **31**, 488‑493 (1941).

[9] Orlova, T., Aßhoff, S. J., Yamaguchi, T., Katsons, N. & Brasselet, E. Creation and manipulation of topological states in chiral nematic microspheres. *Nat. Commun.* **6**, 7603 (2015).

[10] Posnjak, G., Copar, S. & Muševič, I. Points, skyrmions and torons in chiral nematic droplets. *Sci. Rep.* **6**, 26361 (2016).

[11] Posnjak, G., Copar, S. & Muševič, I. Hidden topological constellations and polyvalent charges in chiral nematic droplets. *Nat. Commun.* **8**, 14594 (2017).

[12] Ito, F., Yoshioka, J. & Tabe, Y. Heat-driven rotation in cholesteric droplets with a double twisted structure. *J. Phys. Soc. Jpn.* **85**, 114601 (2016).

[13] Press, M. J. & Arrott, A. S. A. S. Static strain waves in cholesteric liquid-crystals .1. homeotropic boundary-conditions. *J. Phys (Paris)* **37**, 387–395 (1976).

[14] Baudry, J., Brazovskaia, M., Lejcek, L., Oswald, P. & Pirkl, S. Arch-texture in cholesteric liquid crystals. *Liq. Cryst*. **21**, 893–901 (1996).

[15] Smalyukh, I. I. *et al.* Electric-field-induced nematic-cholesteric transition and three-dimensional director structures in homeotropic cells. *Phys. Rev. E* **72**, 061707 (2005).

[16] Seč, D., Čoper, S. & Žumer, S. Topological zoo of free-standing knots in confined chiral nematic fluids. *Nat. Commun.* **5**, 3057 (2014).
