# Supplementary material for: Topology-dependent self-structure mediation and efficient energy conversion in heat-flux-driven rotors of cholesteric droplets
Source: Nat Commun. 2018 Jan 30;9:432. doi: 10.1038/s41467-018-02910-z (PMC5789817; doi:10.1038/s41467-018-02910-z)
Supplement: Supplementary file 3 — Description of Additional Supplementary Files [file 41467_2018_2910_MOESM3_ESM.pdf]

**Description of Additional Supplementary Files**

File Name: Supplementary Movie 1
Description: Ch droplets dispersed in purified water, observed upon subjection to heat-flux of 7.4 $mW \cdot mm^{-2}$ (Movie1.mov). The applied heat-flux was 7.4 $mW \cdot mm^{-2}$. Five times faster video playback speed. Stationary temperature was 45°C. Concentration of the chiral dopant was 2.0 wt%.

File Name: Supplementary Movie 2
Description: Ch droplets dispersed in glycerol, observed upon subjection to heat-flux of 7.4 $mW \cdot mm^{-2}$ (Movie2.mov). Five times faster video playback speed. Stationary temperature was 55°C. Concentration of the chiral dopant was 2.0 wt%.

File Name: Supplementary Movie 3
Description: Ch droplets dispersed in dimethylpolysiloxane (DMPS), observed upon subjection to heat-flux of 7.4 $mW \cdot mm^{-2}$ (Movie3.mov). Five times faster video playback speed. Stationary temperature was 55°C. Concentration of the chiral dopant was 1.0 wt%.

File Name: Supplementary Movie 4
Description: Ch droplets dispersed in poly(perfluoro-4-vinyloxy-3- methyl-1-butene) (CYTOP), observed upon subjection to heat-flux of 7.4 $mW \cdot mm^{-2}$ (Movie4.mov). Five times faster video playback speed. Stationary temperature was 55°C. Concentration of the chiral dopant was 2.0 wt%.

File Name: Supplementary Movie 5
Description: Heat-flux induced rotation in the conventional-type Ch droplets, 'Striped' and 'CC', formed in the coexistence region in the Iso-Ch phase transition (Movie5.mov). Stationary temperature was 55 °C. The applied heat-flux was 6.0 $mW \cdot mm^{-2}$. Real-time playback speed.

File Name: Supplementary Movie 6
Description: Heat-flux induced rotation in the Type-C, D and E3 droplets dispersed in the fluorinated oligomer (Movie6.mov). The concentration of the chiral dopant is 1.0 wt%. The stationary temperature is 55°C. The applied heat-flux is 6.0 $mW \cdot mm^{-2}$. Real-time playback speed.

File Name: Supplementary Movie 7
Description: Heat-flux induced rotation in the Type-E1 and E2 droplets dispersed in the fluorinated oligomer (Movie7.mov). The concentration of the chiral dopant is 2.0 wt%. The stationary temperature is 55°C. The applied heat-flux is 1.5 $mW \cdot mm^{-2}$. Real-time playback speed.

File Name: Supplementary Movie 8
Description: Heat-flux induced rotation in the Type-E3 droplets dispersed in the fluorinated oligomer (Movie8.mov). The concentration of the chiral dopant is 2.0 wt%. The stationary temperature is 55°C. Type-E3 and the unstable state are observed together under the applied heat-flux of 7.4 $mW \cdot mm^{-2}$. Real-time playback speed.
